# Extracellular Polymeric Substances and Biocorrosion/Biofouling: Recent Advances and Future Perspectives

**DOI:** 10.3390/ijms23105566

**Published:** 2022-05-16

**Authors:** Yanan Wang, Ruiyong Zhang, Jizhou Duan, Xin Shi, Yimeng Zhang, Fang Guan, Wolfgang Sand, Baorong Hou

**Affiliations:** 1 CAS Key Laboratory of Marine Environmental Corrosion and Bio-Fouling, Institute of Oceanology, Chinese Academy of Sciences, Qingdao 266071, China; wangyanan@qdio.ac.cn (Y.W.); 17863970979@163.com (X.S.); zhangyimeng21314@163.com (Y.Z.); guanfang1988123@126.com (F.G.); brhou@qdio.ac.cn (B.H.); 2 University of Chinese Academy of Sciences, Beijing 100049, China; 3 Open Studio for Marine Corrosion and Protection, Pilot National Laboratory for Marine Science and Technology (Qingdao), Qingdao 266237, China; 4 Center for Ocean Mega-Science, Chinese Academy of Sciences, Qingdao 266071, China; 5 Institute of Biosciences, University of Mining and Technology, 09599 Freiberg, Germany; wolfgang.sand@uni-due.de; 6 Department of Aquatic Biotechnology, University of Duisburg-Essen, 45141 Essen, Germany; 7 Textile Pollution Controlling Engineering Center of Ministry of Environmental Protection, College of Environmental Science and Engineering, Donghua University, Shanghai 200051, China

**Keywords:** extracellular polymeric substances, microbially influenced corrosion, corrosion protection

## Abstract

Microbial cells secrete extracellular polymeric substances (EPS) to adhere to material surfaces, if they get in contact with solid materials such as metals. After phase equilibrium, microorganisms can adhere firmly to the metal surfaces causing metal dissolution and corrosion. Attachment and adhesion of microorganisms via EPS increase the possibility and the rate of metal corrosion. Many components of EPS are electrochemical and redox active, making them closely related to metal corrosion. Functional groups in EPS have specific adsorption ability, causing them to play a key role in biocorrosion. This review emphasizes EPS properties related to metal corrosion and protection and the underlying microbially influenced corrosion (MIC) mechanisms. Future perspectives regarding a comprehensive study of MIC mechanisms and green methodologies for corrosion protection are provided.

## 1. Introduction

**Figure 1:**
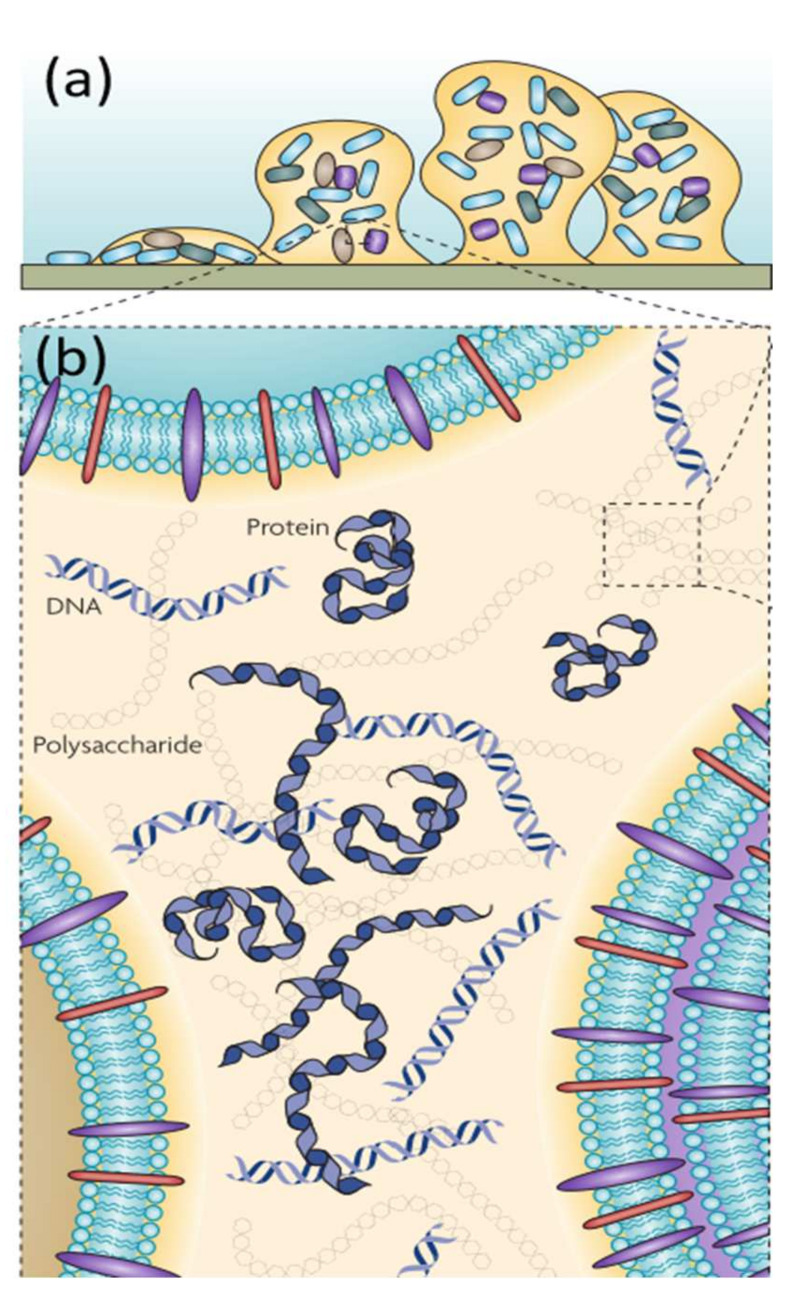
(**a**) The model of a bacterial biofilm attached to a solid surface; biofilm formation begins if bacteria attach to a solid surface and then divide to form microcolonies and produce EPS. Due to bacterial division and EPS formation, different kinds of microorganisms are attracted to enter the consortia/film, and a mature biofilm is gradually formed; (**b**) The major matrix components (polysaccharides, proteins, lipids, and nucleic acid) are mainly distributed between cells, and there are differences in different regions of EPS. Images are from [3]. Reproduced with permission from Springer Nature.

Hydrated biopolymers including polysaccharides, proteins, nucleic acids, and lipids are secreted to encase and immobilize cells. These biopolymers are responsible for the macroscopic appearance of biofilms (Figure 1), which is frequently referred to as “slime” [1]. It is complicated to fully characterize and differentiate EPS composition from cellular components or transiently produced macromolecules. Biochemical and structural identification is hindered by the fact that a diverse array of saccharides with complex linkages are produced, which is defined as “the dark matter of biofilms” [2,3]. EPS were originally abbreviated for extracellular polysaccharides. Later, it was discovered that these so-called extracellular slimes also contain proteins, lipids, humic acids, and other substances. Therefore, EPS were then officially renamed as extracellular polymeric substances [4].

Metal/material corrosion caused by microorganisms attached to the surface of metallic materials is termed as MIC. The problem of MIC causes hundreds of millions of dollars in loss to the global economy every year [5]. Microorganisms secrete EPS to attach and adhere on metal surfaces [3,6]. The interactions of some EPS and biofilms with metals result in metal dissolution. There are many types of microorganisms causing MIC, such as sulfate-reducing prokaryotes (SRP) [7,8,9,10,11,12], manganese/iron-oxidizing bacteria (MOB/IOB) [13,14], iron-reducing bacteria (IRB) [15,16], methanogenic and halophilic archaea [17,18], nitrate-reducing bacteria (NRB) [19,20], and others [21,22]. Table 1 summarizes some representatives of SRP, iron oxidizers/manganese oxidizers, iron reducers, sulfur compound oxidizers, acid producing bacteria/fungi, and microbes that secrete organic acids and produce EPS. Detailed summary is shown in Table 1.

These MIC causing microorganisms are embedded sometimes in an EPS matrix and appear in the form of biofilms [23]. EPS play an important role in the formation of biofilms. They are mainly responsible for the structural and functional integrity of biofilms (Figure 1). The EPS components determine the cohesion, integrity and stress resistance of biofilms and are considered to be the key components determining the physical, chemical, and biological properties of biofilms [3,24]. These properties will determine the corrosion processes of metals. 

**Table 1:** MIC causing microorganisms and their characteristics, summarized and modified on the basis of [25,26].

Type	Aerobic/Anaerobic	Corrosion Agents	Mechanism of Corrosion	References
**Sulfate reducers** *Desulfovibrio* sp. *Desulfomonas* sp. *Desulfotomaculum* *kuznetsovii* *Archaeoglobus fulgidus*	Anaerobic	H_2_S and FeS	Cathodic depolarization by hydrogen uptake, anodic depolarization by corrosive iron sulfides, electrons extracted from Fe^0^	[6,8,11,27,28,29]
**Iron oxidizers/manganese oxidizers** *Gallionella* sp. *Mariprofundus ferrooxydans* *Leptothrix* sp. *Mariprofundus* sp. *Bacillus* sp.	Aerobic	Fe^2+^ to Fe^3+^ and Mn^2+^ to Mn^4+^: Iron oxide and manganese dioxide formation	Deposition of cathodically reactive ferric and manganic oxides	[14,15,30,31,32,33]
**Iron reducers** *Pseudomonas* sp. *Shewanella* sp. *Geobacter sulfurreducens*	Aerobic	Reduce Fe^3+^ to Fe^2+^, Mn^4+^ to Mn^2+^ manganese or iron oxide reduction	Reduction of iron and manganese oxides	[15,31,34]
**Sulfur compound oxidizers** *Thiobacillus* sp. *Acidithiobacillus ferrooxidans* *Acidithiobacillus caldus*	Aerobic	H_2_SO_4_	Acids corrode metal	[35,36,37]
**Acid producing bacteria and fungi** *Clostridium* sp. *Fusarium* sp. *Penicillium* sp. *Hormoconis* sp. *Bacillus subtilis* *Marinobacter* sp.	Aerobic and anaerobic	Acids	Dissolve iron, chelate copper, zinc, and iron	[38,39,40,41,42]
**Slime (EPS) forming bacteria/almost all microorganism** *Clostridium* sp. *Bacillus* sp. *Desulfovibrio* sp. *Pseudomonas* sp.	Aerobic and anaerobic	extracellular polymeric substances (biofilm) or surface compounds/ions	Exopolymers capable of binding metal ions	[43,44,45,46]
**Methanogens** *Methanobacterium* sp. *Methanococcus* sp.	Anaerobic	Extracellar hydrogenases, acids, and CO_2_	Methane production with direct iron oxidation; syntrophic interaction with fermentative microbes or SRP; deposition of cathodically reactive ferric oxides; consumption of hydrogen generated by CO_2_ corrosion	[17,47,48,49]

Current studies on EPS involved in MIC mainly include the analysis of the corrosion behavior of metals in EPS solutions extracted from corrosion-causing microorganisms and exploration of the relationship between EPS and metal corrosion by analyzing surface morphology and composition of metallic materials, environmental parameters, and electrochemical behavior, or by in-vitro simulating the composition of EPS by artificially adding sodium alginate, bovine serum albumin (BSA), and cytochrome *c* to mimic extracellular polysaccharides, proteins, and electrochemically active components [50]. The corrosion effects of EPS produced by various bacteria/archaea on metal corrosion are quite different [51,52]. Existing studies have found that some functional groups in EPS will complex metal ions (iron ions, copper ions, etc.) and, thus, accelerate the dissolution of the anode causing corrosion [53,54]. However, there are also studies showing that the attachment of EPS has an inhibitory effect on the corrosion of a metal, mainly because EPS adsorb on the surface of a material and form a protective film. The negatively charged groups in EPS chelate the metal cations (such as Ca^2+^ and Mg^2+^) in the solution, which results in a dense protective film on the surface of a material, reducing cathodic polarization and, thus, inhibiting corrosion [44,55,56,57]. When copper was immersed in 3.5 wt.% NaCl solution containing EPS produced by an aerotolerant and unidentified *Desulfovibrio*, copper corrosion was promoted in EPS solution for long-term immersion of 11 days, although corrosion was inhibited for a short time of 2 h. Functional residues in the EPS like hydroxyl, carbonyl, carboxyl, and phosphate groups are the main ones to react with Cu, influencing the corrosion behavior of copper in 3.5 wt.% NaCl solution [51]. When 70Cu-30Ni alloy was immersed in EPS produced by *Pseudomonas* NCIMB 2021, a protein-adsorbed metal oxide layer was formed on the surface of 70Cu-30Ni alloy. It inhibited the corrosion of 70Cu-30Ni alloy [58].

To a large extent, microbial corrosion is due to the formation of a biofilm by microorganisms attached to the metal surface. Biofilms are the environment allowing for a survival of microbial cells [3]. Therefore, understanding EPS properties and the role of EPS in the corrosion process is of great significance for knowing the mechanisms of microbial corrosion and for microbial corrosion protection. This review describes EPS analysis, characterization methods, EPS characteristics, and the relationship between EPS and corrosion. In this way, it helps to understand the role of EPS in the corrosion process and provides basic information for corrosion protection.

## 2. EPS Properties

The EPS components determine its structure, function, and reactivity. The diversity of the chemical composition and the properties of EPS play a decisive role in biofilm development and metal corrosion.

### 2.1. Components 

Usually, polysaccharides and proteins make up the major part of microbial EPS, in addition to nucleic acids, humic substances, and lipids as shown in Figure 1b. EPS biochemical composition can be influenced by many factors such as microbial species, substrates, and the downstream extraction methods [59]. The composition of EPS can change during the corrosion process. EPS derived from SRP of *Desulfovibrionaceae* and *Desulfobacteriaceae* species were mainly composed of 60% protein, 37% polysaccharides, and 3% hydrocarbons. Adsorbed EPS on mild steel immersed in the 10-fold diluted EPS solution changed significantly after 60 days; molecular fractions of proteins, polysaccharides and hydrocarbons were changed from 0.64, 0.31, and 0.05 to 0.52, 0.08, and 0.40 [60]. Interestingly, hydrocarbons were detected in EPS materials at the beginning of the experiment. This may be attributed to the used medium based on seawater from Hong Kong Harbor. This harbor was contaminated with hydrocarbons as most harbors are. Most likely, residual hydrocarbons from contaminated seawater were absorbed in EPS. Nevertheless, we believe that conclusion was not justified completely and need a comprehensive investigation (e.g., purification of EPS), or the authors had made a mistake of using hydrocarbon instead of lipids. Functional EPS-groups such as carboxyl and hydroxyl play an important role in bacterial aggregation and biofilm formation [61]. The composition of EPS components changes with the microbial community and the environment, in which microorganisms are located [62,63]. Atalah et al. conducted a characterization of EPS from a thermophilic consortium isolated from a corroded airplane engine and found that the polysaccharides are composed mainly of mannose and glucose residues. The predominant protein was surface (S)-layer protein [64]. The composition of EPS from an unidentified thermophilic SRB strain isolated from the Bohai oilfield in China was dependent on the culture stage. The highest EPS concentrations were extracted from a 14-day old culture [65]. Pressure affects the secretion of EPS. Under high pressure of 35 MPa, *Halanaerobium* spp. secrete more proteins than that of 0.1 MPa probably to increase adhesion [66].

### 2.2. Adhesion

EPS are inherently adhesive. The adhesion not only plays an important role in the formation of biofilms, but also increases the possibility of metal corrosion [67]. Studies described that the adhesion of EPS is related to a S-layer protein, which can act as an adhesive to adjacent cells, thereby further enhancing the aggregation of microorganisms during the formation of biofilms [64,68]. EPS can also increase cell adhesion through hydrophobic forces (primary effect) and/or electrostatic forces (secondary effect) [69]. Acetyl groups are a common substituent of extracellular polysaccharides and increase the adhesion and cohesion of EPS, which may alter the structure of a biofilm [70]. Mayer et al. measured the viscosity of EPS of *Pseudomonas (P.) aeruginosa* using nuclear magnetic resonance (NMR) and viscometry and found that electrostatic interactions and hydrogen bonding were the main reasons for the strong biofilm binding of EPS [71]. Thus, the adhesive properties of EPS increase the accumulation of microorganisms on metal surfaces and the formation of biofilms.

### 2.3. Redox Active EPS and Role in Electron Transfer

**Figure 2:**
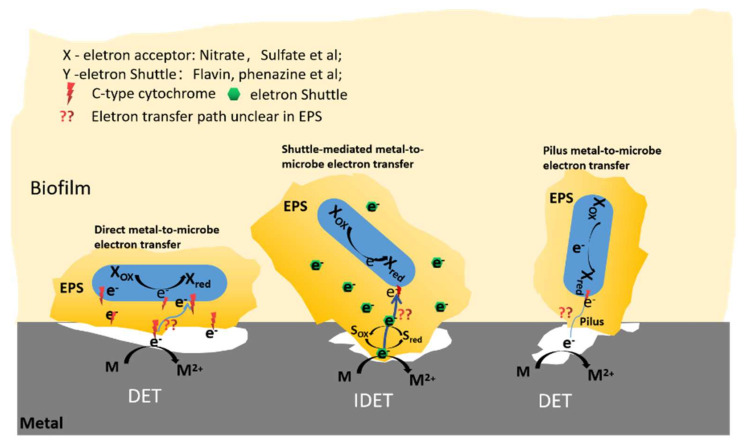
A hypothetical electron transfer pathway in presence and absence of EPS. DET, direct electron transfer; IDET, indirect electron transfer. Adapted from [78].

**Figure 3:**
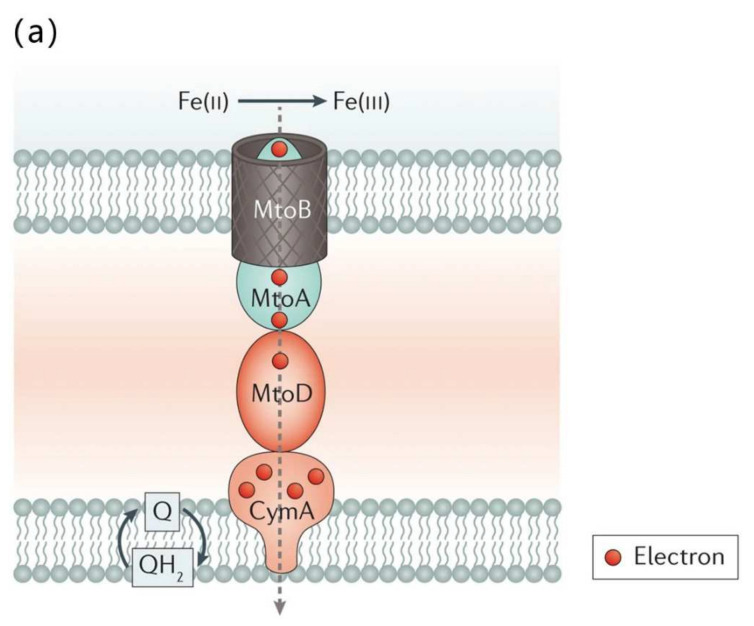

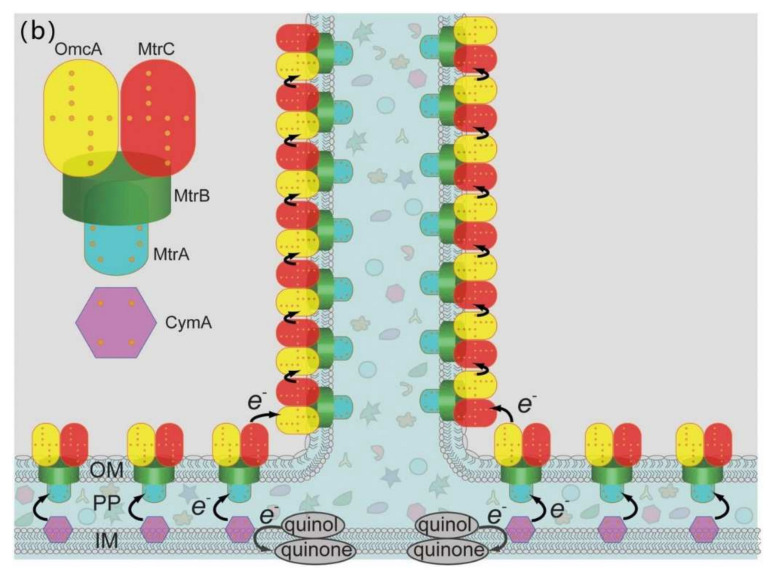
(**a**) *Sideroxydans lithotrophicus* ES-1 uses *c*-type cytochromes (*c*-Cyts) for direct electron transport. The metal oxidation pathway (Mto) of *Sideroxydans lithotrophicus* ES-1 is MtoA (a multihaem *c*-Cyts (mtrA) homolog), MtoB (a porin-like outer membrane protein MtrB homolog), and MtoD (a mono-haem *c*-Cyt) and CymA (a multihaem *c*-Cyt), electrons transferred from extracellular Fe(II) to quinone (Q) in the inner cytoplasmic membrane and cytoplasmic membrane, respectively [72]. Reproduced with permission from Springer Nature. (**b**) Proposed structural model for nanowires of *S. oneidensis* MR-1. *S. oneidensis* MR-1 nanowires are outer membrane (OM) and periplasmic (PP) extension including various *c*-type cytochromes such as OmcA, MtrC, MtrB, MtrA, for electron transfer between these cytochromes, thus enabling extracellular electron transfer in bacteria [79]. Reproduced with permission from PANS.

**Figure 4:**
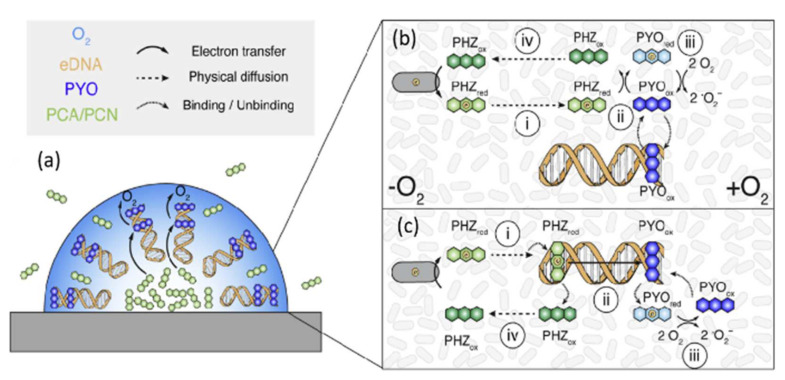
Schematic diagram of eDNA and the electron shuttle compound phenazine bind in biofilm. (**a**) Phenazine binds eDNA to transfer electrons to oxygen in biofilms; (**b**,**c**) two mechanisms of eDNA-mediated phenoazine electron transport [84]. Reproduced with permission from Elsevier.

Many EPS components have been proven to have redox properties and electrochemical activity. Redox active EPS compounds play an important role in the process of electron transfer [72,73]. Extracellular electron transfer is an important component of microbial respiration [74,75]. Metallic materials are used commonly by microorganisms as electron donors [72,76]. Extracellular electron transfer is achieved mainly through direct or indirect electron transfer. The direct electron transfer occurs mainly through *c*-type cytochromes or conductive nanowires, while the indirect electron transfer happens mainly through electron shuttles such as phenazine, humic acids, or flavin (Figure 2). The specific path of bacterial extracellular electron transfer through cytochromes and nanowires is shown in Figure 3 [72,77,78,79]. Membrane-bound cytochrome *c* is a redox protein involved in electron transfer, which contributes often to MIC processes. Through proteomic analysis of the EPS of a *Shewanella* sp. HRCR-1 biofilm, 58 extracellular and outer membrane proteins were identified, including the *c*-type cytochromes Mtr C and Omc A [80]. The electroactive bacteria *Shewanella (S.) oneidensis* contain heme-binding proteins, which are redox components in the EPS [72]. Humic acids are an important component of EPS and can be used as electron shuttle to support microbial growth [81]. In addition, microorganisms can use humic acids as exocellular electron mediator for anaerobic respiration of organic compounds, which is effected by an indirect electron transfer [82]. EPS components can function as electron shuttle in the corrosion process. EPS can combine with iron ions to cause anodic dissolution and promote iron corrosion [54]. EPS can increase the electronic potential and reduce the electronic resistance, thereby increasing the corrosion current by promoting electron transfer and metal dissolution [69]. The metal ions bound in the EPS potentially can act as electron shuttle and transfer electrons to distant microorganisms [83]. Extracellular DNA (eDNA) is also an important component of EPS. Studies have found that eDNA can combine with the electron shuttle compound phenazine to promote the process of extracellular electron transport. Pyocyanin (PYO) and phenazine carboxamide can combine with eDNA to be retained in EPS, thus promoting an effective electron transport (Figure 4) [84].

At present, although it has been proven that some substances in EPS have redox properties, the role of EPS for the corrosion caused by microorganisms using extracellular electron transport remains under debate. The pathway of electron transfer in EPS remains unclear, as shown in Figure 2. Some studies indicate that electron hopping is the most likely hypothesis for electrons to pass through EPS [85]. Studies have also found that EPS polysaccharides can hinder electron transport. These substances remain inactive substances and do not promote the microbial reduction process [50,86].

## 3. EPS Extraction

The choice of EPS extraction greatly affects, qualitatively and quantitatively, the recovery of EPS and its constituents and whether they are obtained in a native state. Therefore, it is very important to choose an appropriate extraction method for studying EPS involved in biocorrosion. The extraction method must avoid the damage of microbial cells. Meanwhile, it is necessary to ensure the extraction efficiency. Up to now, several methods for EPS extraction have been published, as listed below. The specific extraction method is shown in the Supplementary Figure S1.

(1) Physical methods: these methods mainly use an external force to separate EPS from microbial cells and transfer the EPS components to the solution. Commonly used methods include high-speed centrifugation [87], heat extraction [85] and ultrasonic extraction [88]. Among them, the heat extraction may destroy the cell structure of microorganisms due to high temperature application, so that intracellular substances may be released into the EPS solution. This method compromises the EPS purity. Additionally, high temperature may deactivate some redox active substances and enzymes in EPS. Thus, it is necessary to control the temperature and extraction conditions. Dai et al. studied five methods for EPS extraction from *S. oneidensis* MR-1: centrifugation (control), heating (40, 45, 50, and 60 °C), and treatments with H_2_SO_4_, ethylenediaminetetraacetic acid (EDTA), and NaOH. They found for this bacterium that heat (40 °C) and EDTA treatments were the most suitable methods for EPS extraction considering both the low cell lysis and high EPS content [89]. 

(2) Chemical methods: these methods add appropriate amounts of chemical reagents and/or solvents to the cell suspension to extract EPS. Water-soluble EPS macromolecules are solubilized and removed into the extraction medium. Common chemical methods are the use of alkali [90], formaldehyde/NaOH [91], NaCl [92], Ethanol [93], Crown ether [94], EDTA, and cationic resins [91]. The concentration of the extractants should be controlled properly, otherwise cell damage or even disruption may be caused. The cation exchange resin method is the most commonly used one. Sodium ions in the cation exchange resin are removed by divalent cations bound in the EPS of the bacteria. This weakens the forces between the extracellular polymers and the bacterial cell. In the next step, centrifugation as physical force can separate the EPS constituents from the cells [95,96]. 

EPS from *Acidiphilium* 3.2Sup(5) were investigated using five methods: EDTA, NaOH, ion exchange resin, heating, and centrifugation. A high EPS extraction was obtained using EDTA. This method also produced a lesser degree of cellular lysis. Nevertheless, both the amount and the chemical composition of EPS strongly depended on the extraction method used [97]. So far, none of these methods can extract all EPS constituents [98]. Some constituents remain attached to the microbial cells. Consequently, there is no universal EPS extraction method for all microbial EPS type. The extraction process must be adapted to the specific type of biofilm under study and the purpose of the research (Supplementary Table S1).

## 4. EPS Characterization

EPS are a gel, thus their composition and structure cannot be observed directly. Special characterization techniques are required to analyze structure and properties of EPS. These include in-situ observation of the composition of EPS without damaging it.

**Figure 5:**
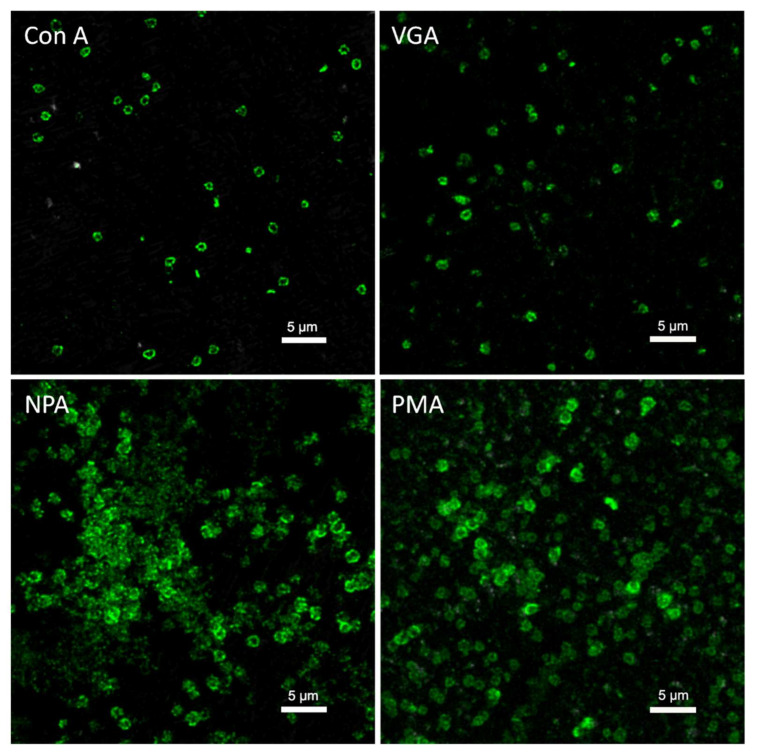
Images of *Sulfolobus metallicus*^T^ biofilms on elemental sulfur stained by Fluorescein isothiocyanate (FITC)-labeled lectins Con A, VGA, NPA (*Narcissus pseudonarcissus*), and PMA (*Polygonatum multiflorum*) are shown. Images were reproduced from [104] with permission from Springer Nature.

One non-destructive method is the use of fluorescently labeled lectins combined with confocal laser scanning microscopy (CLSM) to observe and analyze EPS in biofilms in situ [99]. Lectins are proteins or glycoproteins of plants, animals, or microbial origin. They bind to carbohydrates with a characteristic specificity [100,101]. Fluorescently labelled lectins can be used as probes to investigate an EPS composition enabling the microscopic in situ detection of EPS and its constituents in their distribution in biofilms [102,103,104]. Different types of lectins are used to observe various glycoconjugates in EPS (Figure 5). Different lectins can specifically recognize a specific sugar monomer; for example, the lectin of *Canavalia ensiformis* (Con A) can recognize glucose and mannose residues, the one of *Vicia graminea* (VGA) can recognize n-acetylglucosamine, etc. For details of the lectins see [103]. Strathmann et al. used fluorescently labelled lectins in combination with epifluorescence microscopy and CLSM to visualize and characterize carbohydrate-containing EPS in biofilms of *P. aeruginosa* [101]. Zhang et al. used fluorescently labeled lectins to study the glycoconjugates of EPS secreted by three meso- and thermoacidophilic metal-oxidizing archaea, namely *Ferroplasma acidiphilum*, *Sulfolobus metallicus,* and an unindenfied *Acidianus* sp. on pyrite [103]. They found that EPS of the three archaea are divided into compact and loose EPS. These archaea produced different EPS glycoconjugates on the surface of pyrite [103]. Zippel and Neu et al. characterized the *in* *situ* distribution of EPS glycoconjugates in tufa-associated biofilms of two German hard-water creeks by employing fluorescence lectin-binding analysis [105].

In addition to visual analysis of EPS in biofilms, ^1^H and ^13^C NMR, Fourier transform infrared spectroscopy (FTIR), Raman spectroscopy [98], and X-ray photoelectron spectroscopy (XPS) are used to analyze and identify EPS compounds. The three-dimensional excitation emission matrix (3D-EEM) can also be used to analyze the composition of the EPS. In addition to the above methods used to characterize EPS, there have been many new types of spectroscopic methods for *in situ* characterization of EPS components and functional groups in recent years. Maqbool et al. combined the use of stable isotope analysis and 13C-cross-polarization magic-angle spinning (CPMAS) NMR to examine the extent of the formation of new EPS and the turnover cycles of different carbon structures in EPS [106]. Lin et al. proved carboxyl-rich acidic polysaccharides in EPS by sulfur K-edge X-ray absorption near edge structure (XANES) analysis [107]. Fang et al. used X-ray absorption fine structure (XAFS) spectroscopy to study the adsorption of EPS on goethite extracted from *P. putida* [108]. XAFS results demonstrated that phosphate groups in EPS can form monodentate inner-sphere complexes at pH 3.0, while they form bidentate inner-sphere complexes at pH 9.0 [108]. Scanning transmission X-ray microscopy (STXM)and XAFS are used to characterize the interactions between *Arbuscular mycorrhizal* EPS and Cr ions [109]. STXM allows to obtain EPS structure correlated spectra [110]. The distribution of metal ions with different valence states (such as iron ions) on the surface of EPS can be observed by STXM [86]. EPS at the cell-pyrite interface can be observed by STXM- based C-NEXAFS analysis [111].

## 5. Detection of Cell Lysis 

No matter which method is used for EPS extraction, the extent of cell lysis caused by EPS extraction must be determined and taken into consideration for the validity of the results and conclusions. A harsh extraction of EPS can disrupt the integrity of the cells and lead to a release of intracellular compounds such as polysaccharides and proteins. This is affecting the complexity of the analysis of EPS components and often makes conclusions on the role of EPS constituents impossible. Alginate extracted from *P. aeruginosa* contains uronic acids and alginate does not exist in cells. Therefore, alginate can be used as a sign of contamination with cellular components [112]. N-acetylglucosamine (NAG) is one of the monomers of peptidoglycan in the cell wall. Thus, the degree of cell damage caused by EPS extraction can be judged by detecting the concentration of NAG [113]. The higher the concentration of NAG in the EPS, the more cells have been damaged. Glucose-6-phosphate dehydrogenase (G6PDH) is an enzyme in the cytoplasm of the cell and can be used as a sign of cell lysis [114]. The cell lysis indicated by G6PDH seems to be dependent on the extraction procedure, e.g., stirring intensity, extraction time, dose of the extraction agent [95]. Wang et al. used ATP for quantifying the EPS contamination by intracellular compounds due to cell lysis [115]. The viability of the cells can also be detected by using the LIVE and DEAD staining method to confirm that cells are still active after stripping off the EPS [116]. However, it must be noted that LIVE and DEAD staining regents are only calibrated against the standard cultures and the calculated live/dead ratio may not reveal the true proportion of living/dead cells for these target cultures in each experimental condition (H.-C. Flemming, personal communication). Scanning electron microscopy (SEM) can be used to observe the changes in the morphology of the cells before and after EPS extraction. Atomic force microscopy (AFM) can also be used to observe the morphology of microorganisms before and after EPS extraction [85,86]. The determination of cell damage aims mainly to detect intracellular substances outside of cells to judge about possible contamination of the EPS extraction. According to the needs of an actual test, the detection of cell lysis needs to be selected. In addition to the detection of landmark substances, AFM and SEM can be combined to detect and observe the activity and morphology of bacteria after extracting EPS.

## 6. EPS and Biocorrosion 

At present, it can be ascertained that EPS can accelerate or inhibit corrosion depending on the microorgansims and environments. The following chapter is divided into two parts to discuss the relationships between EPS and biocorrosion.

### 6.1. EPS Accelerated Corrosion

The acceleration of metal corrosion caused by EPS mainly includes the following points: (1) EPS contain a large number of anionic groups such as carboxyl, hydroxyl and/or amino groups, etc. These groups can chelate metal ions, and thus promote the occurrence of corrosion [117]; Copper metal was corroded after 11 days of immersion in EPS extracted from the sulfate reducing bacterium *Desulfovibrio* sp. Copper corrosion is promoted due to the destruction of the protective Cu_2_O film by EPS and its constituents. The main reason causing corrosion was that the hydroxyl, carbonyl, carboxyl, and phosphate groups in EPS reacted with Cu^+^ in the Cu_2_O protective film, which destroyed it finally [51]. The functional groups in the EPS play an important role for the shuttling electrons in the corrosion process of iron metals. EPS combined with iron ions lead to anodic dissolution promoting Fe corrosion [54]. EPS can increase the electric potential and reduce the electric resistance of materials causing an increase of the corrosion current. Thereby, they are promoting electron transfer and metal dissolution [52]. If microorganisms come into contact with metal surfaces, EPS have been shown to chelate iron ions and stimulate the interaction between microorganisms and metal ions [83]; (2) EPS can change the chemical properties of corrosion products and their morphology, and promote the corrosion of metals [68]; When a metal is immersed in EPS extracted from an iron-oxidizing bacterium, accelerated corrosion was detected [52]. Arkan et al. found that *Desulfovibrio* sp. led to the pitting corrosion of 316L SS, mainly due to the production of extracellular proteins [118]. Li et al. performed electrochemical and surface analyses for mild steel and pure copper and found that EPS accumulated on the metal surface. This resulted in an heterogenous biofilm and the formation of oxygen concentration cell causing corrosion, leading to severe pitting [119]. Zhang et al. analysed the in situ microbial diversity in artificial surface-associated marine biofilms and found that EPS-related genes were more abundant in copper-associated biofilms than that in aluminium-associated biofilms. They thus proposed that copper corrosion in natural marine environments was attributed to the heterogeneous microenvironments within the biofilms caused by the EPS [120]. Fang et al. found that EPS increased the corrosion of mild steel due to its acidity and its binding with iron [121]. Interestingly, it was reported that EPS production of *Halanaerobium congolense* WG8 increased, if the hydraulic pressure increased. Consequently, MIC was accelerated [66]. This study points out the necessity of studying microbial physiology and MIC under high pressure conditions such as hydraulically fractured shales or deep-sea environments; (3) When corrosion of metals is accelerated with biofilm formation and growth, anodic dissolution can occur. The metallic dissolution would be accelerated to form complexes with EPS. Consequently, the over-potential of metallic dissolution would decrease, and mixed potential should be shifted in the less noble direction, although Little et al. repeatedly described the potential shift in nobilization in the noble direction as an important characteristic for MIC [122,123]. When a metal is immersed in EPS extracted from unidentified iron-oxidizing bacteria isolated from the sludge of a Sinopec oilfield in China, accelerated corrosion was detected. It was found by potentiodynamic polarization curve that the corrosion current was higher and the corrosion potential was lower in the solution containing EPS in comparison with that containing no EPS, and EPS promoted the cathodic reaction processes [52].

### 6.2. EPS Inhibit Corrosion

**Figure 6:**
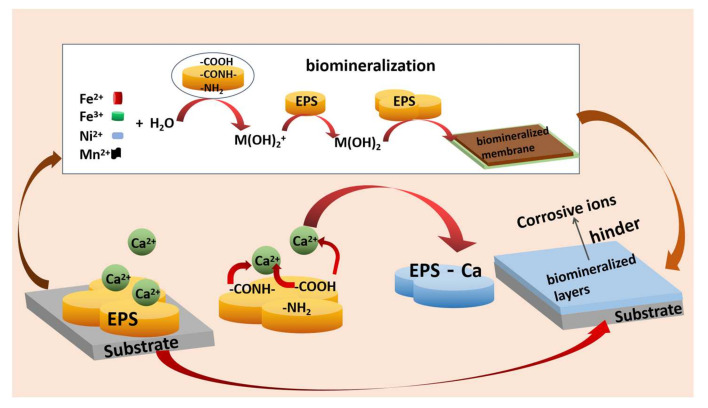
Diagram of formation of a biomineralized film. Adapted from [56]. Reproduced with permission from Elsevier.

EPS can adsorb on the metal surface and form a biofilm, which acts as a protective layer, hinders the transmission of oxygen, and separates a metal surface from a corrosive environment, thus, metal corrosion is inhibited [53,54,124,125,126,127]. The negatively charged functional groups in EPS, such as -NH_2_, -COOH, -C-O-C, and -OH, first adsorb positively charged metal ions to form complexes. Then, EPS interact with metal ions by oxidation and precipitation to form biomineralized layers on the metal surface, which can protect metals from corrosive environments such as seawater [56,128,129]. The EPS control the kinetic pathway of CaCO_3_, biomineralizing nucleation and CaCO_3_ crystal growth. The formation mechanism of a biomineralized film is illustrated in Figure 6 [56,130]. Microorganisms with an over-production of EPS form a biomineralization film on the surface of a metal and protect it from corrosion. However, if microorganisms secrete only a small amount of EPS, a complete biological mineralization cannot be achieved. Consequently, accelerated corrosion due to the formation of concentrated elements on the surface will occur [130]. The researchers extracted polysaccharides from EPS and showed that the hydroxyl groups in the extracellular polysaccharides can adsorb/chelate Fe^2+^/Fe^3+^ ions and attach to the surface of carbon steel. In this way, a dense protective film is formed, which reduces the diffusion rate of oxygen and Cl^-^ ions and, thus, inhibits the corrosion of carbon steel [131,132]. Li et al. found that EPS can protect X65 steel by preventing bacterial cells of SRP from adhering to the surface of X65 steel and blocking SRP from gaining electrons from the metal [133]. Recently, it was shown that a marine isolate of *P. stutzeri* can promote the formation of a protective biomineralization film, which had a good inhibition effect against steel corrosion [134]. *S. putrefaciens* could use cell walls as the nucleation sites to induce the formation of a protective biomineralized layer, which contained calcite and EPS on the steel surface [129]. 

In summary, the role of EPS in corrosion depends mainly on: (1) whether the functional groups in the EPS can interact with metal ions; (2) whether the film formed by EPS adsorption on the metal surface is closed or patchy to isolate the metal from the corrosive environment; (3) whether EPS components such as proteins and/or humic acids can carry out electron transfer and are related to the redox state of metals. In any case, the influence of EPS and its component for the corrosion of metals need to be explored further.

### 6.3. Application of EPS as a Corrosion Inhibitor

EPS can be used as a new type of “green” corrosion inhibitor for metals [44]. They can be adsorbed on the metal surface and complexed with metal ions to form a protective film. This film acts as a barrier isolating a metal surface from a corrosive environment. Thereby the reaction between cathode and the anode is prevented. Moradi et al. found that EPS produced by *Vibrio neocaledonicus* can inhibit the corrosion of carbon steel in seawater up to ±95% [57]. The EPS extracted from waste-activated sludge also has a strong inhibitory effect on carbon steel, which is comparable to commercial corrosion inhibitors [124]. EPS secreted by a new isolate of *Marinobacter aquaeolei* has a corrosion inhibition efficiency of more than 91% for X80 steel, that proves that these EPS are an effective corrosion inhibitor [125]. As a corrosion inhibitor, an admixture based on EPS 180 exopolysaccharides was used in coatings. It has a good inhibitory effect on the corrosion of reinforced concrete. As a corrosion inhibitor, EPS will not affect the water absorption of concrete [135]. Differently functionalised cyclodextrins as EPS-analogue substances were tested to evaluate their inhibition effect on MIC by *Desulfovibrio vulgaris* under anaerobic conditions [136]. Out of the 25 tested compounds, (polymerised) carboxy(m)ethylated cyclodextrins showed the highest protective effect against MIC with 77% lower corrosion rates after 21 d incubation. 

## 7. Perspective and Future Directions 

New technical methods have been applied to explore the relationship between microorganisms and EPS and metal corrosion. The gene knockout method can control and adjust EPS secretion and verify the functional roles of EPS components [130]. Bioinformatics, mutational studies, and transcriptional profiling of biofilms have been used for identification of additional EPS components. Wu et al. identified multiple genes of *Pandoraea* sp. XY-2 involved in exopolysaccharide synthesis and EPS formation by bioinformatic analysis [137]. Cao et al. used a global proteomic approach and identified a total of 58 extracellular and outer membrane proteins in the EPS [80]. Twenty redox proteins were found in extracted EPS, such as *c*-type cytochromes, Mtr C, and Omc A, which have been implicated to participate in extracellular electron transfer [80,138]. The use of fluorescent markers such as lectins and CLSM can realize the visual and in situ analysis of EPS [103,139]. Lectins can quickly identify carbohydrates in EPS and can be used to determine the role of EPS in microbial corrosion.

The study of EPS relevant to MIC is mainly limited in laboratory studies by the use of pure cultures. The data may not reflect the complexity of MIC processes. The mechanism of metal corrosion in natural biofilm environments is unclear still. Due to the complexity of EPS components and the diversity of EPS functions, the role EPS play in the process of corrosion requires further research. Improved knowledge regarding the relationship between biofilms/EPS and metal corrosion will provide further insights into the mechanism and the prevention of MIC.

## Supplementary Materials

The following supporting information can be downloaded at: https://www.mdpi.com/article/10.3390/ijms23105566/s1.

## References

[1] Flemming H.-C., Wingender J., Szewzyk U., Steinberg P., Rice S.A., Kjelleberg S. (2016). Biofilms: An emergent form of bacterial life. Nat. Rev. Microbiol..

[2] Flemming H.-C., Neu T.R., Wozniak D.J. (2007). The EPS matrix: The House of Biofilm cells. J. Bacteriol..

[3] Flemming H.-C., Wingender J. (2010). The biofilm matrix. Nat. Rev. Microbiol..

[4] Wingender J., Neu T.R., Flemming H.-C., Wingender J., Neu T.R., Flemming H.-C. (1999). What are Bacterial Extracellular Polymeric Substances?. Microbial Extracellular Polymeric Substances: Characterization, Structure and Function.

[5] Hou B., Li X., Ma X., Du C., Zhang D., Zheng M., Xu W., Lu D., Ma F. (2017). The cost of corrosion in China. Npj Mater. Degrad..

[6] Wikiel A.J., Datsenko I., Vera M., Sand W. (2014). Impact of *Desulfovibrio alaskensis* biofilms on corrosion behaviour of carbon steel in marine environment. Bioelectrochemistry.

[7] Xu L., Guan F., Ma Y., Zhang R., Zhang Y., Zhai X., Dong X., Wang Y., Duan J., Hou B. (2022). Inadequate dosing of THPS treatment increases microbially influenced corrosion of pipeline steel by inducing biofilm growth of *Desulfovibrio hontreensis* SY-21. Bioelectrochemistry.

[8] Enning D., Venzlaff H., Garrelfs J., Dinh H.T., Meyer V., Mayrhofer K., Hassel A.W., Stratmann M., Widdel F. (2012). Marine sulfate-reducing bacteria cause serious corrosion of iron under electroconductive biogenic mineral crust. Environ. Microbiol..

[9] Xu D., Li Y., Gu T. (2016). Mechanistic modeling of biocorrosion caused by biofilms of sulfate reducing bacteria and acid producing bacteria. Bioelectrochemistry.

[10] Liu H., Meng G., Li W., Gu T., Liu H. (2019). Microbiologically influenced corrosion of carbon steel beneath a deposit in CO_2_-saturated formation water containing *Desulfotomaculum nigrificans*. Front. Microbiol..

[11] Amin O.A., Aragon E., Fahs A., Davidson S., Ollivier B., Hirschler-Rea A. (2020). Iron corrosion induced by the hyperthermophilic sulfate-reducing archaeon *Archaeoglobus fulgidus* at 70 °C. Int. Biodeterior. Biodegrad..

[12] Jia R., Yang D., Xu D., Gu T. (2018). Carbon steel biocorrosion at 80 °C by a thermophilic sulfate reducing archaeon biofilm provides evidence for its utilization of elemental iron as electron donor through extracellular electron transfer. Corros. Sci..

[13] Yue Y., Lv M., Du M. (2019). The corrosion behavior and mechanism of X65 steel induced by iron-oxidizing bacteria in the seawater environment. Mater. Corros.-Werkst. Korros..

[14] Thyssen C., Holuscha D., Kuhn J., Walter F., Fürbeth W., Sand W. (2015). Biofilm formation and stainless steel corrosion analysis of *Leptothrix discophora*. Adv. Mater. Res..

[15] Lee J.S., McBeth J.M., Ray R.I., Little B.J., Emerson D. (2013). Iron cycling at corroding carbon steel surfaces. Biofouling.

[16] Hernandez-Santana A., Kokbudak H.N., Nanny M.A. (2022). The influence of iron-binding ligands in the corrosion of carbon steel driven by iron-reducing bacteria. Npj Mater. Degrad..

[17] Lahme S., Mand J., Longwell J., Smith R., Enning D. (2021). Severe corrosion of carbon steel in oil field produced water can be linked to methanogenic archaea containing a special type of [NiFe] hydrogenase. Appl. Environ. Microbiol..

[18] Qian H., Ju P., Zhang D., Ma L., Hu Y., Li Z., Huang L., Lou Y., Du C. (2019). Effect of dissolved oxygen concentration on the microbiologically influenced corrosion of Q235 carbon steel by halophilic archaeon *Natronorubrum tibetense*. Front. Microbiol..

[19] Lahme S., Enning D., Callbeck C.M., Vega D.M., Curtis T.P., Head I.M., Hubert C.R.J., Stams A.J.M. (2019). Metabolites of an oil field sulfide-oxidizing, nitrate-reducing *Sulfurimonas* sp. cause severe corrosion. Appl. Environ. Microbiol..

[20] Jia R., Yang D., Xu D., Gu T. (2017). Anaerobic corrosion of 304 stainless steel caused by the *Pseudomonas aeruginosa* biofilm. Front. Microbiol..

[21] Imo E., Orji J., Nweke C. (2018). Influence of *Aspergillus fumigatus* on corrosion behaviour of mild steel and aluminium. Int. J. Appl. Microbiol. Biotechnol. Res..

[22] Zhang Y., Zhai X., Guan F., Dong X., Sun J., Zhang R., Duan J., Zhang B., Hou B. (2022). Microbiologically influenced corrosion of steel in coastal surface seawater contaminated by crude oil. Npj Mater. Degrad..

[23] Tuck B., Watkin E., Somers A., Machuca L.L. (2022). A critical review of marine biofilms on metallic materials. Npj Mater. Degrad..

[24] Flemming H.C., Wingender J. (2001). Relevance of microbial extracellular polymeric substances (EPSs)—Part I: Structural and ecological aspects. Water Sci. Technol..

[25] Kip N., van Veen J.A. (2015). The dual role of microbes in corrosion. ISME J..

[26] Enning D., Garrelfs J. (2014). Corrosion of iron by sulfate-reducing bacteria: New views of an old problem. Appl. Environ. Microbiol..

[27] Lan X., Zhang J., Wang Z., Zhang R., Sand W., Zhang L., Duan J., Zhu Q., Hou B. (2022). Corrosion of an AZ31B magnesium alloy by sulfate-reducing prokaryotes in a mudflat environment. Microorganisms.

[28] Venzlaff H., Enning D., Srinivasan J., Mayrhofer K.J.J., Hassel A.W., Widdel F., Stratmann M. (2013). Accelerated cathodic reaction in microbial corrosion of iron due to direct electron uptake by sulfate-reducing bacteria. Corros. Sci..

[29] Anandkumar B., Choi J.H., Venkatachari G., Maruthamuthu S. (2009). Molecular characterization and corrosion behavior of thermophilic (55 degrees C) SRB *Desulfotomaculum kuznetsovii* isolated from cooling tower in petroleum refinery. Mater. Corros.-Werkst. Und Korros..

[30] McBeth J.M., Little B.J., Ray R.I., Farrar K.M., Emerson D. (2011). Neutrophilic iron-oxidizing “Zetaproteobacteria” and mild steel corrosion in nearshore marine environments. Appl. Environ. Microbiol..

[31] Rao T.S., Sairam T.N., Viswanathan B., Nair K.V.K. (2000). Carbon steel corrosion by iron oxidising and sulphate reducing bacteria in a freshwater cooling system. Corros. Sci..

[32] Chen S., Deng H., Liu G., Zhang D. (2019). Corrosion of Q235 carbon steel in seawater containing *Mariprofundus ferrooxydans* and *Thalassospira* sp.. Front. Microbiol..

[33] Linhardt P. (2010). Twenty years of experience with corrosion failures caused by manganese oxidizing microorganisms. Mater. Corros.-Werkst. Korros..

[34] Tang H.-Y., Holmes D.E., Ueki T., Palacios P.A., Lovley D.R. (2019). Iron corrosion via direct metal-microbe electron transfer. Mbio.

[35] Li S.-M., Zhang Y.-Y., Liu J.-H., Yu M. (2008). Corrosion behavior of steel A3 influenced by *Thiobacillus ferrooxidans*. Acta Phys.-Chim. Sin..

[36] Inaba Y., West A.C., Banta S. (2020). Enhanced microbial corrosion of stainless steel by *Acidithiobacillus ferrooxidans* through the manipulation of substrate oxidation and over expression of rus. Biotechnol. Bioeng..

[37] Dong Y., Jiang B., Xu D., Jiang C., Li Q., Gu T. (2018). Severe microbiologically influenced corrosion of S32654 super austenitic stainless steel by acid producing bacterium *Acidithiobacillus caldus* SM-1. Bioelectrochemistry.

[38] Ramos Monroy O.A., Ruiz Ordaz N., Hernandez Gayosso M.J., Juarez Ramirez C., Galindez Mayer J. (2019). The corrosion process caused by the activity of the anaerobic sporulated bacterium *Clostridium celerecrescens* on API XL 52 steel. Environ. Sci. Pollut. Res..

[39] Juzeliunas E., Ramanauskas R., Lugauskas A., Leinartas K., Samuleviciene M., Sudavicius A., Juskenas R. (2007). Microbially influenced corrosion of zinc and aluminium—Two-year subjection to influence of *Aspergillus niger*. Corros. Sci..

[40] Little B., Staehle R., Davis R. (2001). Fungal influenced corrosion of post-tensioned cables. Int. Biodeterior. Biodegrad..

[41] Usher K.M., Kaksonen A.H., MacLeod I.D. (2014). Marine rust tubercles harbour iron corroding archaea and sulphate reducing bacteria. Corros. Sci..

[42] Wang Y.S., Liu L., Fu Q., Sun J., An Z.Y., Ding R., Li Y., Zhao X.D. (2020). Effect of *Bacillus subtilis* on corrosion behavior of 10MnNiCrCu steel in marine environment. Sci. Rep..

[43] Jia R., Yang D., Xu J., Xu D., Gu T. (2017). Microbiologically influenced corrosion of C1018 carbon steel by nitrate reducing *Pseudomonas aeruginosa* biofilm under organic carbon starvation. Corros. Sci..

[44] Stadler R., Fuerbeth W., Harneit K., Grooters M., Woellbrink M., Sand W. (2008). First evaluation of the applicability of microbial extracellular polymeric substances for corrosion protection of metal substrates. Electrochim. Acta.

[45] San N.O., Nazir H., Donmez G. (2011). Microbial corrosion of Ni-Cu alloys by *Aeromonas eucrenophila* bacterium. Corros. Sci..

[46] Stadler R., Wei L., Fuerbeth W., Grooters M., Kuklinski A. (2010). Influence of bacterial exopolymers on cell adhesion of *Desulfovibrio vulgaris* on high alloyed steel: Corrosion inhibition by extracellular polymeric substances (EPS). Mater. Corros.-Werkst. Korros..

[47] Kato S. (2016). Microbial extracellular electron transfer and its relevance to iron corrosion. Microb. Biotechnol..

[48] Deutzmann J.S., Sahin M., Spormann A.M. (2015). Extracellular enzymes facilitate electron uptake in biocorrosion and bioelectrosynthesis. Mbio.

[49] Liang B., Wang L.-Y., Mbadinga S.M., Liu J.-F., Yang S.-Z., Gu J.-D., Mu B.-Z. (2015). *Anaerolineaceae* and *Methanosaeta* turned to be the dominant microorganisms in alkanes-dependent methanogenic culture after long-term of incubation. Amb Express.

[50] Gao Y., Trueman B.F., Stoddart A.K., Gagnon G.A. (2018). Understanding the impact of extracellular polymeric substances on lead release in drinking water systems. Acs Omega.

[51] Chen S., Zhang D. (2018). Study of corrosion behavior of copper in 3.5 wt.% NaCl solution containing extracellular polymeric substances of an aerotolerant sulphate-reducing bacteria. Corros. Sci..

[52] Liu H., Gu T., Asif M., Zhang G., Liu H. (2017). The corrosion behavior and mechanism of carbon steel induced by extracellular polymeric substances of iron-oxidizing bacteria. Corros. Sci..

[53] Dong Y.H., Guo N., Liu T., Dong L.H., Yin Y.S. (2016). Effect of extracellular polymeric substances isolated from *Vibrio natriegens* on corrosion of carbon steel in seawater. Corros. Eng. Sci. Technol..

[54] Jin J., Guan Y. (2014). The mutual co-regulation of extracellular polymeric substances and iron ions in biocorrosion of cast iron pipes. Bioresour. Technol..

[55] Jin J., Wu G., Zhang Z., Guan Y. (2014). Effect of extracellular polymeric substances on corrosion of cast iron in the reclaimed wastewater. Bioresour. Technol..

[56] Li S., Qu Q., Li L., Xia K., Li Y., Zhu T. (2019). *Bacillus cereus* s-EPS as a dual bio-functional corrosion and scale inhibitor in artificial seawater. Water Res..

[57] Moradi M., Song Z., Xiao T. (2018). Exopolysaccharide produced by *Vibrio neocaledonicus* sp as a green corrosion inhibitor: Production and structural characterization. J. Mater. Sci. Technol..

[58] Bautista B.E.T., Wikiel A.J., Datsenko I., Vera M., Sand W., Seyeux A., Zanna S., Frateur I., Marcus P. (2015). Influence of extracellular polymeric substances (EPS) from *Pseudomonas* NCIMB 2021 on the corrosion behaviour of 70Cu-30Ni alloy in seawater. J. Electroanal. Chem..

[59] Flemming H.-C., Neu T., Wingender J. (2016). The Perfect Slime-and the ‘Dark Matter’ of Biofilms.

[60] Chan K.Y., Xu L.C., Fang H.H.P. (2002). Anaerobic electrochemical corrosion rug of mild steel in the presence of extracellular polymeric substances produced by a culture enriched in sulfate-reducing bacteria. Environ. Sci. Technol..

[61] Hou X., Liu S., Zhang Z. (2015). Role of extracellular polymeric substance in determining the high aggregation ability of anammox sludge. Water Res..

[62] Fagerlund A., Langsrud S., Heir E., Mikkelsen M.I., Moretro T. (2016). Biofilm matrix composition affects the susceptibility of food associated *Staphylococci* to cleaning and disinfection agents. Front. Microbiol..

[63] Rehman Z.U., Rehm B.H.A. (2013). Dual roles of *Pseudomonas aeruginosa* AlgE in secretion of the virulence factor alginate and formation of the secretion complex. Appl. Environ. Microbiol..

[64] Atalah J., Blamey L., Gelineo-Albersheim I., Blamey J.M. (2019). Characterization of the EPS from a thermophilic corrosive consortium. Biofouling.

[65] Dong Z.H., Liu T., Liu H.F. (2011). Influence of EPS isolated from thermophilic sulphate-reducing bacteria on carbon steel corrosion. Biofouling.

[66] Booker A.E., Hoyt D.W., Meulia T., Eder E., Nicora C.D., Purvine S.O., Daly R.A., Moore J.D., Wunch K., Pfiffner S.M. (2019). Deep-subsurface pressure stimulates metabolic plasticity in shale-colonizing *Halanaerobium* spp.. Appl. Environ. Microbiol..

[67] Geesey G.G., Jang L., Jolley J.G., Hankins M.R., Iwaoka T., Griffiths P.R. (1988). Binding of metal-ions by extracellular polymers of biofilm bacteria. Water Sci. Technol..

[68] Cheng S., Lau K.-T., Chen S., Chang X., Liu T., Yin Y. (2010). Microscopical observation of the marine bacterium *Vibrio natriegeus* growth on metallic corrosion. Mater. Manuf. Processes.

[69] Wang J., Tian B., Bao Y., Qian C., Yang Y., Niu T., Xin B. (2018). Functional exploration of extracellular polymeric substances (EPS) in the bioleaching of obsolete electric vehicle LiNi(x)Co(y)Mn1-O-x-y(2) Li-ion batteries. J. Hazard. Mater..

[70] Tielen P., Strathmann M., Jaeger K.E., Flemming H.C., Wingender J. (2005). Alginate acetylation influences initial surface colonization by mucoid *Pseudomonas aeruginosa*. Microbiol. Res..

[71] Mayer C., Moritz R., Kirschner C., Borchard W., Maibaum R., Wingender J., Flemming H.C. (1999). The role of intermolecular interactions: Studies on model systems for bacterial biofilms. Int. J. Biol. Macromol..

[72] Shi L., Dong H., Reguera G., Beyenal H., Lu A., Liu J., Yu H.-Q., Fredrickson J.K. (2016). Extracellular electron transfer mechanisms between microorganisms and minerals. Nat. Rev. Microbiol..

[73] Ye J., Hu A., Ren G., Chen M., Tang J., Zhang P., Zhou S., He Z. (2018). Enhancing sludge methanogenesis with improved redox activity of extracellular polymeric substances by hematite in red mud. Water Res..

[74] Gralnick J.A., Newman D.K. (2007). Extracellular respiration. Mol. Microbiol..

[75] Lovley D.R. (2008). Extracellular electron transfer: Wires, capacitors, iron lungs, and more. Geobiology.

[76] Tang H.-Y., Yang C., Ueki T., Pittman C.C., Xu D., Woodard T.L., Holmes D.E., Gu T., Wang F., Lovley D.R. (2021). Stainless steel corrosion via direct iron-to-microbe electron transfer by *Geobacter* species. ISME J..

[77] Gu T., Jia R., Unsal T., Xu D. (2019). Toward a better understanding of microbiologically influenced corrosion caused by sulfate reducing bacteria. J. Mater. Sci. Technol..

[78] Lekbach Y., Liu T., Li Y., Moradi M., Dou W., Xu D., Smith J.A., Lovley D.R. (2021). Microbial corrosion of metals: The corrosion microbiome. Adv. Microb. Physiol..

[79] Pirbadian S., Barchinger S.E., Leung K.M., Byun H.S., Jangir Y., Bouhenni R.A., Reed S.B., Romine M.F., Saffarini D.A., Shi L. (2014). *Shewanella oneidensis* MR-1 nanowires are outer membrane and periplasmic extensions of the extracellular electron transport components. Proc. Natl. Acad. Sci. USA.

[80] Cao B., Shi L., Brown R.N., Xiong Y., Fredrickson J.K., Romine M.F., Marshall M.J., Lipton M.S., Beyenal H. (2011). Extracellular polymeric substances from *Shewanella* sp. HRCR-1 biofilms: Characterization by infrared spectroscopy and proteomics. Environ. Microbiol..

[81] Lovley D.R., Coates J.D., BluntHarris E.L., Phillips E.J.P., Woodward J.C. (1996). Humic substances as electron acceptors for microbial respiration. Nature.

[82] Stams A.J.M., de Bok F.A.M., Plugge C.M., van Eekert M.H.A., Dolfing J., Schraa G. (2006). Exocellular electron transfer in anaerobic microbial communities. Environ. Microbiol..

[83] Beech W.B., Sunner J. (2004). Biocorrosion: Towards understanding interactions between biofilms and metals. Curr. Opin. Biotechnol..

[84] Saunders S.H., Tse E.C.M., Yates M.D., Otero F.J., Trammell S.A., Stemp E.D.A., Barton J.K., Tender L.M., Newman D.K. (2020). Extracellular DNA promotes eficient extracellular electron transfer by pyocyanin in *Pseudomonas aeruginosa* biofilms. Cell.

[85] Xiao Y., Zhang E., Zhang J., Dai Y., Yang Z., Christensen H.E.M., Ulstrup J., Zhao F. (2017). Extracellular polymeric substances are transient media for microbial extracellular electron transfer. Sci. Adv..

[86] Gao L., Lu X., Liu H., Li J., Li W., Song R., Wang R., Zhang D., Zhu J. (2019). Mediation of extracellular polymeric substances in microbial reduction of hematite by *Shewanella oneidensis* MR-1. Front. Microbiol..

[87] Fang H.H.P., Jia X.S. (1996). Extraction of extracellular polymer from anaerobic sludges. Biotechnol. Tech..

[88] Dignac M.F., Urbain V., Rybacki D., Bruchet A., Snidaro D., Scribe P. (1998). Chemical description of extracellular polymers: Implication on activated sludge floc structure. Water Sci. Technol..

[89] Dai Y.-F., Xiao Y., Zhang E.-H., Liu L.-D., Qiu L., You L.-X., Mahadevan G.D., Chen B.-L., Zhao F. (2016). Effective methods for extracting extracellular polymeric substances from *Shewanella oneidensis* MR-1. Water Sci. Technol..

[90] Brown M.J., Lester J.N. (1980). Comparison of bacterial extracellular polymer extraction methods. Appl. Environ. Microbiol..

[91] Liu H., Fang H.H.P. (2002). Extraction of extracellular polymeric substances (EPS) of sludges. J. Biotechnol..

[92] Christensen B.E., Kjosbakken J., Smidsrod O. (1985). Partial chemical and physical characterization of two extracellular polysaccharides produced by marine, periphytic *Pseudomonas* sp strain NCMB-2021. Appl. Environ. Microbiol..

[93] Forster C.F., Clarke A.R. (1983). The production of polymer from activated-sludge by ethanolic extraction and its relation to treatment-plant operation. Water Pollut. Control.

[94] Wuertz S., Spaeth R., Hinderberger A., Grieba T., Flemming H.C., Wilderer P.A. (2001). A new method for extraction of extracellular polymeric substances from biofilms and activated sludge suitable for direct quantification of sorbed metals. Water Sci. Technol..

[95] Frolund B., Palmgren R., Keiding K., Nielsen P.H. (1996). Extraction of extracellular polymers from activated sludge using a cation exchange resin. Water Res..

[96] Jahn A., Nielsen P.H. (1995). Extraction of extracellular polymeric substances (EPS) from biofilms using a cation exchange resin. Water Sci. Technol..

[97] Tapia J., Munoz J., Gonzalez F., Blázquez M., Malki M., Ballester A. (2009). Extraction of extracellular polymeric substances from the acidophilic bacterium *Acidiphilium* 3.2 Sup (5). Water Sci. Technol..

[98] Pronk M., Neu T.R., van Loosdrecht M.C.M., Lin Y.M. (2017). The acid soluble extracellular polymeric substance of aerobic granular sludge dominated by *Defluviicoccus* sp.. Water Res..

[99] Neu T.R., Lawrence J.R., Donelli G. (2014). Advanced techniques for in situ analysis of the biofilm matrix (structure, composition, dynamics) by means of laser scanning microscopy. Microbial Biofilms.

[100] Neu T.R., Lawrence J.R., Abelson J., Simon M. (1999). Lectin-binding analysis in biofilm systems. Methods in Enzymology.

[101] Strathmann M., Wingender J., Flemming H.C. (2002). Application of fluorescently labelled lectins for the visualization and biochemical characterization of polysaccharides in biofilms of *Pseudomonas aeruginosa*. J. Microbiol. Methods.

[102] Lawrence J.R., Swerhone G.D.W., Kuhlicke U., Neu T.R. (2007). In Situ evidence for microdomains in the polymer matrix of bacterial microcolonies. Can. J. Microbiol..

[103] Zhang R.Y., Neu T.R., Bellenberg S., Kuhlicke U., Sand W., Vera M. (2015). Use of lectins to in situ visualize glycoconjugates of extracellular polymeric substances in acidophilic archaeal biofilms. Microb. Biotechnol..

[104] Zhang R., Neu T.R., Zhang Y., Bellenberg S., Kuhlicke U., Li Q., Sand W., Vera M. (2015). Visualization and analysis of EPS glycoconjugates of the thermoacidophilic archaeon *Sulfolobus metallicus*. Appl. Microbiol. Biotechnol..

[105] Zippel B., Neu T.R. (2011). Characterization of glycoconjugates of extracellular polymeric substances in Tufa-associated biofilms by using fluorescence lectin-binding analysis. Appl. Environ. Microbiol..

[106] Maqbool T., Cho J., Shin K.H., Hur J. (2020). Using stable isotope labeling approach and two dimensional correlation spectroscopy to explore the turnover cycles of different carbon structures in extracellular polymeric substances. Water Res..

[107] Lin H., Wang C., Zhao H., Chen G., Chen X. (2020). A subcellular level study of copper speciation reveals the synergistic mechanism of microbial cells and EPS involved in copper binding in bacterial biofilms. Environ. Pollut..

[108] Fang L., Cao Y., Huang Q., Walker S.L., Cai P. (2012). Reactions between bacterial exopolymers and goethite: A combined macroscopic and spectroscopic investigation. Water Res..

[109] Wu S., Zhang X., Sun Y., Wu Z., Li T., Hu Y., Lv J., Li G., Zhang Z., Zhang J. (2016). Chromium immobilization by extra- and intraradical fungal structures of arbuscular mycorrhizal symbioses. J. Hazard. Mater..

[110] Liu H.-C., Xia J.-L., Nie Z.-Y., Zhen X.-J., Zhang L.-J. (2015). Differential expression of extracellular thiol groups of moderately thermophilic *Sulfobacillus thermosulfidooxidans* and extremely thermophilic *Acidianus manzaensis* grown on S^0^ and Fe^2+^. Arch. Microbiol..

[111] Mitsunobu S., Zhu M., Takeichi Y., Ohigashi T., Suga H., Jinno M., Makita H., Sakata M., Ono K., Mase K. (2016). Direct detection of Fe(II) in extracellular polymeric substances (EPS) at the mineral-microbe interface in bacterial pyrite leaching. Microbes Environ..

[112] Wingender J., Strathmann M., Rode A., Leis A., Flemming H.C., Doyle R.J. (2001). Isolation and biochemical characterization of extracellular polymeric substances from *Pseudomonas aeruginosa*. Microbial Growth in Biofilms, Pt A: Developmental and Molecular Biological Aspects.

[113] Nagata T., Meon B., Kirchman D.L. (2003). Microbial degradation of peptidoglycan in seawater. Limnol. Oceanogr..

[114] Ng F.M.W., Dawes E.A. (1973). Chemostat studies on regulation of glucose-metabolism in *Pseudomonas-aeruginosa* by citrate. Biochem. J..

[115] Wang S., Redmile-Gordon M., Mortimer M., Cai P., Wu Y., Peacock C.L., Gao C., Huang Q. (2019). Extraction of extracellular polymeric substances (EPS) from red soils (Ultisols). Soil Biol. Biochem..

[116] Boulos L., Prevost M., Barbeau B., Coallier J., Desjardins R. (1999). LIVE/DEAD (R) BacLight (TM): Application of a new rapid staining method for direct enumeration of viable and total bacteria in drinking water. J. Microbiol. Methods.

[117] Sheng G.-P., Yu H.-Q. (2007). Formation of extracellular polymeric substances from acidogenic sludge in H_2_-producing process. Appl. Microbiol. Biotechnol..

[118] Arkan S., Ilhan-Sungur E., Cansever N. (2016). Corrosive metabolic activity of *Desulfovibrio* sp. on 316L stainless steel. J. Mater. Eng. Perform..

[119] Li X.L., Narenkumar J., Rajasekar A., Ting Y.-P. (2018). Biocorrosion of mild steel and copper used in cooling tower water and its control. 3 Biotech.

[120] Zhang Y., Ma Y., Zhang R., Zhang B., Zhai X., Li W., Xu L., Jiang Q., Duan J., Hou B. (2019). Metagenomic resolution of functional diversity in copper surface-associated marine biofilms. Front. Microbiol..

[121] Fang H.H.P., Xu L.C., Chan K.Y. (2002). Effects of toxic metals and chemicals on biofilm and biocorrosion. Water Res..

[122] Little B.J., Lee J.S. (2014). Microbiologically influenced corrosion: An update. Int. Mater. Rev..

[123] Little B.J., Hinks J., Blackwood D.J. (2020). Microbially influenced corrosion: Towards an interdisciplinary perspective on mechanisms. Int. Biodeterior. Biodegrad..

[124] Go L.C., Holmes W., Depan D., Hernandez R. (2019). Evaluation of extracellular polymeric substances extracted from waste activated sludge as a renewable corrosion inhibitor. Peerj.

[125] Khan M.S., Yang C., Zhao Y., Pan H., Zhao J., Shahzad M.B., Kolawole S.K., Ullah I., Yang K. (2020). An induced corrosion inhibition of X80 steel by using marine bacterium *Marinobacter salsuginis*. Colloids Surf. B Biointerfaces.

[126] Li Z., Zhou J., Yuan X., Xu Y., Xu D., Zhang D., Feng D., Wang F. (2021). Marine biofilms with significant corrosion inhibition performance by secreting extracellular polymeric substances. ACS Appl. Mater. Interfaces.

[127] Gao Y., Zhang M., Fan Y., Li Z., Cristiani P., Chen X., Xu D., Wang F., Gu T. (2022). Marine *Vibrio* spp. protect carbon steel against corrosion through secreting extracellular polymeric substances. Npj Mater. Degrad..

[128] Guo Z., Pan S., Liu T., Zhao Q., Wang Y., Guo N., Chang X., Liu T., Dong Y., Yin Y. (2019). *Bacillus subtilis* inhibits *Vibrio natriegens*-induced corrosion via biomineralization in seawater. Front. Microbiol..

[129] Lou Y., Chang W., Cui T., Qian H., Huang L., Ma L., Hao X., Zhang D. (2021). Microbiologically influenced corrosion inhibition of carbon steel via biomineralization induced by *Shewanella putrefaciens*. Npj Mater. Degrad..

[130] Liu T., Guo Z., Zeng Z., Guo N., Lei Y., Liu T., Sun S., Chang X., Yin Y., Wang X. (2018). Marine bacteria provide lasting anticorrosion activity for steel via biofilm-induced mineralization. Acs Appl. Mater. Interfaces.

[131] Ghafari M.D., Bahrami A., Rasooli I., Arabian D., Ghafari F. (2013). Bacterial exopolymeric inhibition of carbon steel corrosion. Int. Biodeterior. Biodegrad..

[132] Finkenstadt V.L., Cote G.L., Willett J.L. (2011). Corrosion protection of low-carbon steel using exopolysaccharide coatings from *Leuconostoc mesenteroides*. Biotechnol. Lett..

[133] Li Q., Wang J., Xing X., Hu W. (2018). Corrosion behavior of X65 steel in seawater containing sulfate reducing bacteria under aerobic conditions. Bioelectrochemistry.

[134] Liu H., Chen W., Tan Y., Meng G., Liu H., Cheng Y., Liu H. (2022). Characterizations of the biomineralization film caused by marine *Pseudomonas stutzeri* and its mechanistic effects on X80 pipeline steel corrosion. J. Mater. Sci. Technol..

[135] Roux S., Bur N., Ferrari G., Tribollet B., Feugeas F. (2010). Influence of a biopolymer admixture on corrosion behaviour of steel rebars in concrete. Mater. Corros.-Werkst. Korros..

[136] Kuklinski A. (2017). Development of Extracellular Polymeric Substance-Derived Protective Films Against Microbiologically Influenced Corrosion by *Desulfovibrio vulgaris*. Ph.D. Thesis.

[137] Wu X., Wu X., Zhou X., Gu Y., Zhou H., Shen L., Zeng W. (2020). The roles of extracellular polymeric substances of *Pandoraea* sp. XY-2 in the removal of tetracycline. Bioprocess Biosyst. Eng..

[138] Zhou E., Li F., Zhang D., Xu D., Li Z., Jia R., Jin Y., Song H., Li H., Wang Q. (2022). Direct microbial electron uptake as a mechanism for stainless steel corrosion in aerobic environments. Water Res..

[139] Zhang R., Neu T.R., Blanchard V., Vera M., Sand W. (2019). Biofilm dynamics and EPS production of a thermoacidophilic bioleaching archaeon. New Biotechnol..

