# Supplementary material for: Extracellular Polymeric Substances and Biocorrosion/Biofouling: Recent Advances and Future Perspectives
Source: Int J Mol Sci. 2022 May 16;23(10):5566. doi: 10.3390/ijms23105566 (PMC9143384; doi:10.3390/ijms23105566)
Supplement: Supplementary file 1 [file ijms-23-05566-s001.zip › ijms-1682569-supplementary.pdf]

International Journal of *Molecular Sciences*

MDPI

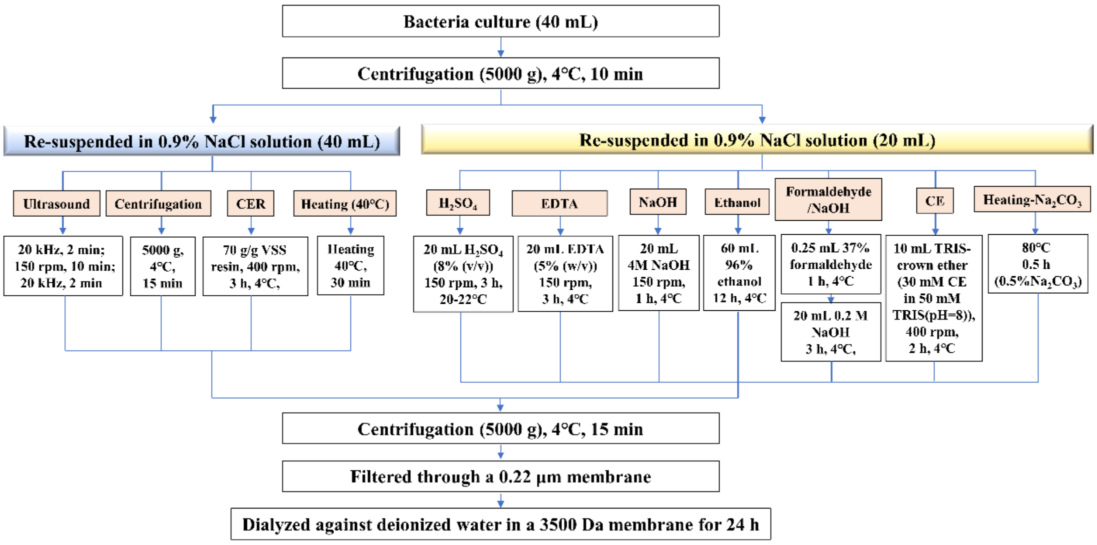

Figure S1. Procedures of different EPS extraction methods.

Table S1. Comparison of different EPS extraction methods.

Extraction Method	Extraction Sources	Degree of cell lysis	EPS content compared to centrifugation and ultrasound#	Ref.
Heating (40 °C)	*Shewanella oneidensis* MR-1/*Geobacter sulfurreducens* PCA and *Geobacter soli* GSS01	Low	High	[1,2]
H_2SO_4	*Shewanella oneidensis* MR-1/anaerobic ammonium oxidation	High	Relatively high	[1,3]
EDTA	*Shewanella oneidensis* MR-1/activated sludge/anaerobic ammonium oxidation/*Geobacter sulfurreducens* PCA and *Geobacter soli* GSS01	Low	Relatively high (humic substance and DNA)	[1,2,4]
NaOH	*Shewanella oneidensis* MR-1	High	Relatively high	[1]
Formaldehyde/ NaOH	activated sludge/anaerobic ammonium oxidation	Low	Relatively high（carbohydrate, protein and uronic acid）	[3,4]
CER	*Shewanella oneidensis* MR-1/activated sludge/anaerobic ammonium oxidation	Low	high	[3–5]
CE	activated sludge	Low	Relatively high（carbohydrate, protein and uronic acid）	[4]
Ethanol	activated sludge	Low	Relatively low	[4]
Heating -Na_2CO_3	anaerobic ammonium oxidation	Low	High (proteins)	[3]

#: In parentheses are the relative benefits of the method for extracting certain components of EPS.

References

1. Dai, Y.-F.; Xiao, Y.; Zhang, E.-H.; Liu, L.-D.; Qiu, L.; You, L.-X.; Mahadevan, G. D.; Chen, B.-L.; Zhao, F., Effective methods for extracting extracellular polymeric substances from *Shewanella oneidensis* MR-1. *Water Sci. Technol.* **2016,** *74*, 2987-2996.

2. Yang, G. Q.; Lin, J.; Zeng, E. Y.; Zhuang, L., Extraction and characterization of stratified extracellular polymeric substances in Geobacter biofilms. *Bioresour. Technol.* **2019,** *276*, 119-126.
3. Feng, C. J.; Lotti, T.; Lin, Y. M.; Malpei, F., Extracellular polymeric substances extraction and recovery from anammox granules: Evaluation of methods and protocol development. *Chem. Eng. J.* **2019,** *374*, 112-122.
4. Zhang, L.; Geng, J.; Ding, L.; Ren, H., Inorganic fractions in extracellular polymeric substance extracted from activated sludge and biofilm samples by different methods. *Water Sci. Technol.* **2012,** *66*, 1203-1211.
5. Li, S.-W.; Sheng, G.-P.; Cheng, Y.-Y.; Yu, H.-Q., Redox properties of extracellular polymeric substances (EPS) from electroactive bacteria. *Sci. Rep.* **2016,** *6*, 39098.
